# Whom tuberculosis tests detect and why it matters: implications for diagnostic algorithms

**DOI:** 10.1016/j.lanmic.2025.101237

**Published:** 2025-10-17

**Authors:** Emily A Kendall, Claudia M Denkinger, Adithya Cattamanchi, David W Dowdy, Jason R Andrews

**Affiliations:** Division of Infectious Diseases, Johns Hopkins University School of Medicine, Baltimore, MD, USA; Department of Epidemiology, Johns Hopkins Bloomberg School of Public Health, Baltimore, MD, USA; Division of Infectious Disease and Tropical Medicine, Heidelberg University Hospital, Heidelberg, Germany; German Center of Infection Research, partner site Heidelberg, Heidelberg, Germany; Division of Pulmonary Diseases and Critical Care Medicine, University of California Irvine, Irvine, CA, USA; Center for Tuberculosis, Institute for Global Health Sciences, University of California San Francisco, San Francisco, CA, USA; Department of Epidemiology, Johns Hopkins Bloomberg School of Public Health, Baltimore, MD, USA; Division of Infectious Diseases and Geographic Medicine, Stanford University, Stanford, CA, USA

## Abstract

Tuberculosis encompasses a spectrum of characteristics—including bacillary burden, clinical severity, and access to care—that are relevant to clinical and epidemiological outcomes and the performance of diagnostic assays. The value of diagnostic assays depends not only on their numerical accuracy, which can vary substantially between populations, but also on which individuals with and without tuberculosis the assays identify. Moreover, detectable features of tuberculosis, such as pathogen burden or host responses, are often correlated, making it difficult to predict the accuracy and impact of diagnostic algorithms from the accuracies of individual component tests. Therefore, when evaluating novel tuberculosis diagnostics, greater consideration should be given to characterising which segments of the disease spectrum are detected, how these segments overlap across tests, and how they are prioritised for detection. Understanding these relationships is particularly crucial for screening, given that screening seeks to detect a broad spectrum of disease and often uses multistep algorithms. We present a framework for understanding the sensitivity and specificity of assays and algorithms as the degree of alignment between different subsets of the disease spectrum. Based on this framework, we make recommendations for the measurement, reporting, target setting, and interpretation of diagnostic accuracy to guide both novel test development and the optimal use of existing diagnostics.

## Introduction

In the ongoing efforts to reduce the persistently high global burden of tuberculosis, limitations in diagnostics remain a major barrier. People might live with tuberculosis for a year or more, often spending many months seeking care for symptoms before diagnosis,^1^ and an estimated 25% of tuberculosis cases are never diagnosed or notified.^2^ A major reason for the inadequacy of current diagnostic assays is that tuberculosis is not a homogeneous entity but exists across a multidimensional spectrum. Tuberculosis can range from entirely extrapulmonary disease to forms that are readily detectable by sputum smear microscopy.^3^ Clinically, a large proportion of people with prevalent tuberculosis are asymptomatic, whereas others have symptoms that range from mild to debilitating.^4^ Immunologically, responses to *Mycobacterium tuberculosis* are highly robust in some people and nearly imperceptible in others.^5,6^ Socially, some people with tuberculosis have ready access to high-quality diagnostic infrastructure, whereas others face substantial barriers to accessing care.^7^ Many of these dimensions are also affected by comorbidities and age.

Numerous efforts are under way—some with promising early results—to develop assays that make tuberculosis screening and diagnosis more accessible. These include approaches such as testing closer to the point-of-care, using more accessible specimen types, reducing assay and platform costs, and improving detection of extrapulmonary and paucibacillary tuberculosis.^8^ However, much of the current thinking and guidance on these novel assays remains anchored in numerical estimates of accuracy (ie, sensitivity and specificity) that implicitly ignore the multidimensional spectrum of tuberculosis. For example, recently updated target product profiles (TPPs) for tuberculosis diagnostic tests^9^ and a newly planned TPP for screening tests^10^ acknowledge different use cases but still set invariant numerical targets for sensitivity and specificity. Similarly, the ability to meet fixed sensitivity and specificity benchmarks has been a primary focus in early clinical evaluations of potential screening or diagnostic tests, such as digital chest x-ray,^11^ C-reactive protein,^12,13^ tongue swab molecular testing,^14,15^ and cough sound signatures.^16^ Numerical benchmarks for accuracy can obscure the dependence of diagnostic outcomes on the population and context in which a test is used and on other tests with which it is paired.

As efforts to detect tuberculosis become more proactive and extend to a broader range of health-care and community settings, they encounter a broader spectrum of disease and use an expanding array of novel tests and potential multitest algorithms.^9,17^ With these developments, it is increasingly important to understand accuracy in a way that translates to diverse populations, test combinations, and diagnostic objectives. In this Personal View, we argue that it is helpful to interpret a sensitivity or specificity estimate as a degree of alignment between the results of the assay in question and those of other assays or population selection mechanisms with which the index assay is paired—set against the backdrop of the underlying disease spectrum. Subsequently, we identify the best practices for characterising, reporting, and modelling test accuracies and their correlations, with the aim of improving the understanding of test performance in relation to the disease spectrum and facilitating the design of improved testing algorithms and the development of more impactful tests. Although these considerations apply to tuberculosis diagnosis in any context, they are particularly relevant for population-based screening, where the disease spectrum is particularly broad, testing algorithms are typically multistep, and much of the available data on test performance are extrapolated from accuracy studies in symptomatic, care-seeking populations. Despite the fact that our focus is on tests and algorithms for diagnosing tuberculosis, many of the considerations discussed also apply to assays for latent *M tuberculosis* infection and to other diseases with heterogeneous phenotypes and diagnostic uncertainty.

## Test sensitivities and specificities represent proportions of a disease spectrum

The sensitivity and specificity of a diagnostic assay are often conceptualised as random probabilities, whereby a homogeneous pool of people, differing only by the presence or absence of disease, are assigned positive or negative test results by chance. However, in reality, people lie along a spectrum of detectability determined by their disease characteristics. Setting aside laboratory sources of variation, diagnostic tests identify individuals whose disease features exceed a particular threshold of detectability. This threshold—rather than any numerical estimate of sensitivity and specificity—represents the true characteristic of the diagnostic assay. For any given assay, the threshold of detectability influences its accuracy across different populations, its ability to complement other tests, and its clinical and epidemiological impact.

**Figure 1:**
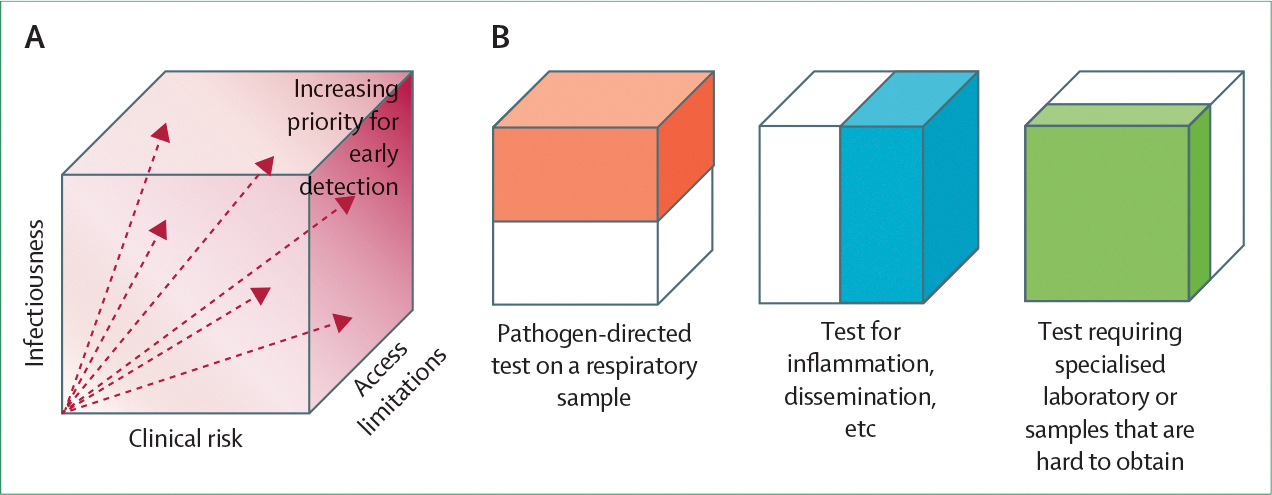
Dimensions of the tuberculosis spectrum and their interactions with diagnostic or screening tests (A) Dimensions of the tuberculosis spectrum that might align with the objectives of diagnostic development and testing: detecting tuberculosis cases that are highly infectious, at high risk of poor clinical outcomes if not detected promptly, and difficult to diagnose using existing tests and testing processes. Access limitations encompass physical access to care, societal barriers to care, or the ability to readily obtain appropriate clinical specimens. (B) Any given test is likely to detect only some dimensions of this spectrum. Tests that detect pathogen in the respiratory tract are likely to be positive in individuals with the highest respiratory pathogen burden, corresponding to a greater infectious potential. Tests that detect a disseminated pathogen or host inflammatory response might identify individuals at an elevated risk for poor clinical outcomes. Although access factors are not captured in most estimates of sensitivity, tests located in central laboratories or requiring difficult-to-obtain samples might have low reach or yield and miss individuals with limited access to health care.

The spectrum of detectability typically aligns with one or more dimensions of the tuberculosis disease spectrum (figure 1). For example, tuberculosis can be associated with a range of bacterial burdens, varying degrees of anatomical localisation versus dissemination, and differing degrees of host inflammatory response. Many tuberculosis diagnostic tests—including sputum microscopy and culture, various sputum rapid molecular tests, and new tongue swab-based molecular assays^18^—are designed to detect *M tuberculosis* in the respiratory tract. The sensitivity of these assays depends on the proportion of people with tuberculosis whose respiratory *M tuberculosis* burden exceeds the test’s limit of detection. Other assays detect host responses to *M tuberculosis*, including non-specific inflammatory responses (eg, C-reactive protein)^19^ specific antigen recognition (eg, interferon-gamma response assays, which are mainly useful in diagnosing exposure and infection^20^), or transcriptomic signatures with some degree of specificity for both tuberculosis and active disease status.^21^ The sensitivity of tests based on these biomarkers depends on the proportion of people with tuberculosis whose host responses exceed a threshold level. Other dimensions influence the spectrum of detectability—for example, chest x-ray^22,23^ detects macroscopic pathological changes and urine lipoarabinomannan (LAM) detects bacterial components at other anatomical sites.^24,25^ Beyond purely biological factors, the ability to access health care (influenced by social and economic factors) and the ability to provide a diagnostic specimen (eg, sputum) are additional dimensions that identify which people with tuberculosis are detected by a given test.

## Different segments of the disease spectrum have different health consequences

An ideal diagnostic test would detect everyone with tuberculosis (and exclude everyone without the disease). Nonetheless, real-world accuracies are imperfect and frequently conflict with other objectives. For example, highly sensitive laboratory-based tests might have less reach than less sensitive point-of-care tests.^18^ Therefore, when setting accuracy targets, it is important to consider whether some people with tuberculosis should be prioritised for detection. Specifically, detecting the same number of people with tuberculosis could have different clinical and public health benefits depending on the characteristics of those detected—including their transmission potential, risk of death or serious clinical sequelae if missed, and access to care (figure 1).

For example, assays that detect *M tuberculosis* in the respiratory tract might be particularly useful in active case-finding campaigns aimed at limiting (airborne) transmission within communities. By contrast, tests that detect disseminated bacteria (eg, urine LAM) or measure non-specific but potentially deleterious host responses (eg, C-reactive protein) might be more effective in identifying people with advanced disease who will benefit clinically from early diagnosis and treatment.

## Correlated interactions with the disease spectrum in multistep algorithms

Tests are often used in combination, with diagnostic outcomes and impact depending on the combined performance of multiple assays. When diagnosing tuberculosis in symptomatic patients, complementary tests could be combined in parallel to improve sensitivity (eg, concurrent use of a sputum molecular test and urine LAM^26^). In tuberculosis screening among individuals not seeking care for symptoms, multitest algorithms are particularly common, with tests typically applied sequentially (as screening and confirmatory steps) to improve specificity.^17^ A new TPP for tuberculosis screening tests also considers algorithms with multiple sequential screening steps.^10^

As test accuracy reflects the ability to detect disease manifestations, different tuberculosis tests often yield correlated results. The correlation is particularly strong when the tests target similar dimensions of disease; for example, assays designed to detect *M tuberculosis* in the respiratory tract have varying sensitivities but highly correlated results.^14,27,28^ Even among tests that detect seemingly unrelated disease manifestations, substantial correlations can exist, reflecting different dimensions of the disease process. For example, despite considerable heterogeneity, individuals with a higher *M tuberculosis* burden in sputum tend, on average, to also have more advanced lung pathology and stronger host responses. Illustrative studies have shown that these correlations result in higher sensitivity of non-sputum-based screening tests among people with higher sputum bacillary burden than among people with lower sputum bacillary burden (as categorised by semiquantitative Xpert MTB/RIF Ultra [Xpert]): 97% versus 69% for a transcriptomic host-response signature,^29^ 91% versus 47% for a standard C-reactive protein cutoff,^13^ and 96% versus 82% for an artificial intelligence-based chest x-ray interpretation algorithm.^30^

**Figure 2:**
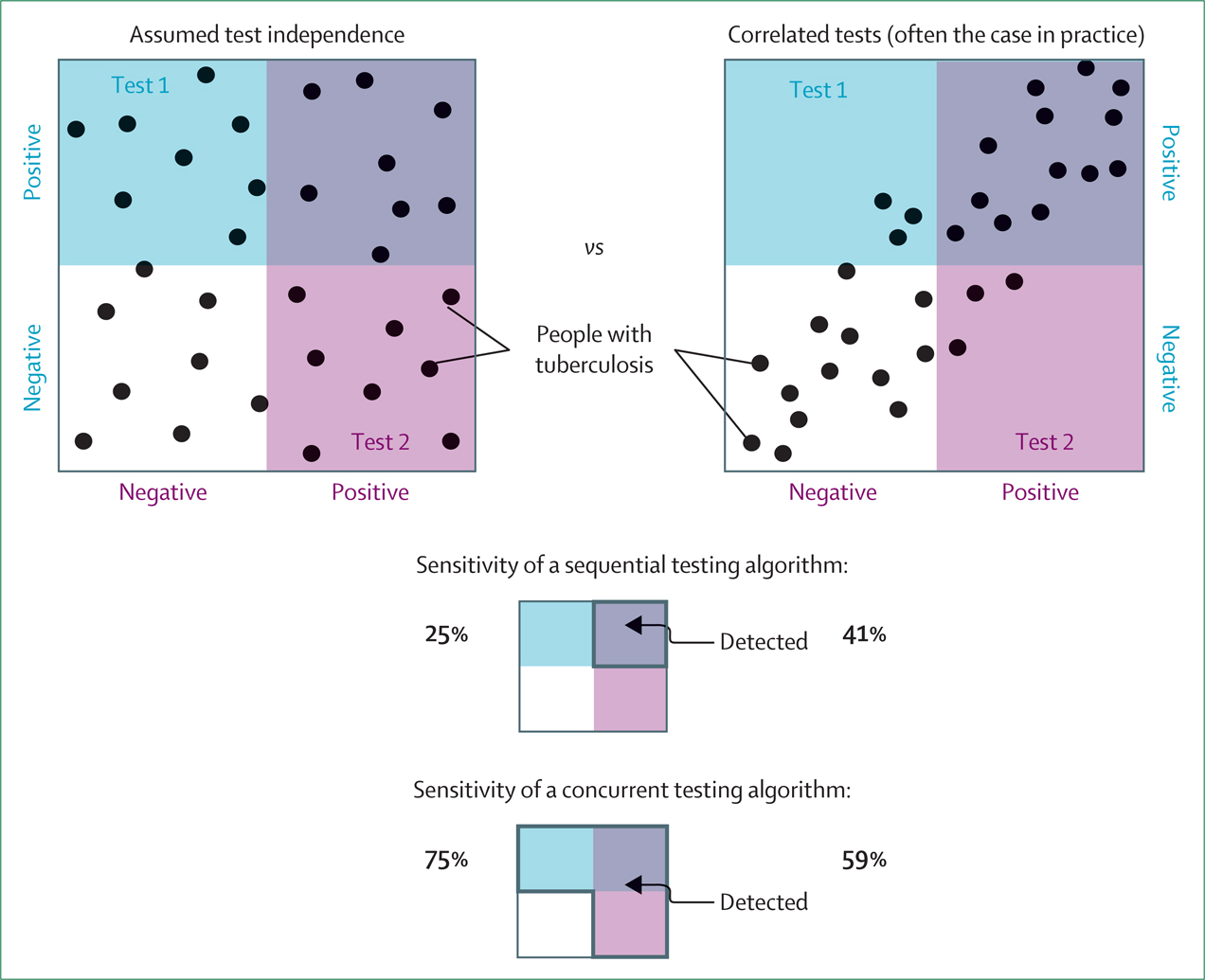
Measurement of shared or correlated dimensions of disease often leads to correlation between test results Squares represent the possible spectrum of tuberculosis along two dimensions corresponding to the horizontal and vertical axes. Disease is evenly distributed across both disease dimensions, and people with tuberculosis are indicated by black dots. Test 1 detects the 50% of individuals with the highest values along the vertical dimension (dots within the blue shaded area), while Test 2 detects the 50% of individuals with the highest values along the horizonal dimension (dots within the purple shaded area). If the two dimensions are uncorrelated (left), the sensitivity of a sequential testing algorithm—where a second test is applied if the first is positive, as is typical of screening algorithms—is equal to the product of the individual test sensitivities (25%). However, often, individuals at an extreme of one disease dimension are more likely to also lie at a similar extreme along other disease dimensions (eg, bacterial burden, symptoms, and inflammatory markers tend to be correlated). This correlation between tests increases the sensitivity of sequential testing algorithms (from 25% to 41% [13 of 32] of tuberculosis cases falling in the top-right quadrant detected by both tests in this illustration). Similar correlations reduce the sensitivity of concurrent or parallel testing algorithms, in which a positive result on either test is a positive algorithm result (reduced from 75% to 59% in this illustration). Correlations have opposite effects on specificity (not shown).

These correlations can be an important determinant of the overall accuracy of test combinations. Although independence for each testing step is often assumed, positive correlations increase the sensitivity and reduce the specificity of sequential testing algorithms while exerting opposite effects in concurrent testing (figure 2). For example, a common case-finding approach is to screen based on symptoms or chest x-ray or both, followed by confirmatory sputum testing. Among individuals with culture-positive tuberculosis in representative prevalence surveys, 42% screened positive for symptoms and 41% were smear positive.^31^ If sputum smear results and symptoms were independent, one would expect 17% (0·42 × 0·41) of people with tuberculosis to be both symptomatic and smear positive. However, owing to modest correlations between smear status and symptoms, this percentage was 22%—approximately 1·3 times higher.^31^ Therefore, when evaluating tests for use in algorithms, it is important to characterise correlations among different tests that might be used in combination.

## Correlations determine spectrum bias and the importance of reference standards

Similar to screening tests in sequential testing algorithms, the processes that select populations for testing often preferentially include individuals from particular segments of the disease spectrum. This selection can lead to diagnostic spectrum bias, the magnitude of which depends on the degree of overlap between the segment of the spectrum represented in the testing population and the segment detectable by the test. The potential for spectrum bias is particularly high in tuberculosis, because common approaches to selecting diagnostic study populations—such as enrolling individuals who present to clinics or hospitals with tuberculosis-like symptoms—tend to be strongly correlated with dimensions of the disease spectrum, including bacterial burden and inflammatory response, that are used in diagnosis. Consequently, sensitivity might decline substantially when tests are applied to less symptomatic populations, such as in community-based screening.

Although the reference standard used for comparison does not affect the actual clinical performance of a test, it affects the evaluation of that performance and can strongly influence estimates of the test’s accuracy. More restrictive reference standards—particularly if they are correlated with the test under evaluation—might result in higher numerical sensitivity estimates. Such dependence is particularly important for tuberculosis, given the difficulty of defining a reference standard that accurately classifies the full microbiological^32^ and clinical^33^ heterogeneity of the disease. Therefore, it might be advantageous to align the reference standard with the segment of the disease spectrum that an assay is intended to detect. Depending on the circumstances, a sputum culture-based reference standard could be either too broad or too restrictive. For example, a sputum culture-based reference standard might underestimate the accuracy of an assay (eg, urine LAM) whose added value lies primarily in its ability to detect extrapulmonary or disseminated disease that is often sputum culture-negative. Conversely, for assays intended to be followed in practice by a confirmatory test less sensitive than culture (eg, a screening test that will be followed by sputum molecular confirmation), comparison to culture might unduly penalise otherwise high-value assays for failing to detect paucibacillary tuberculosis that would have been missed by the molecular confirmatory test regardless.

**Figure 3:**
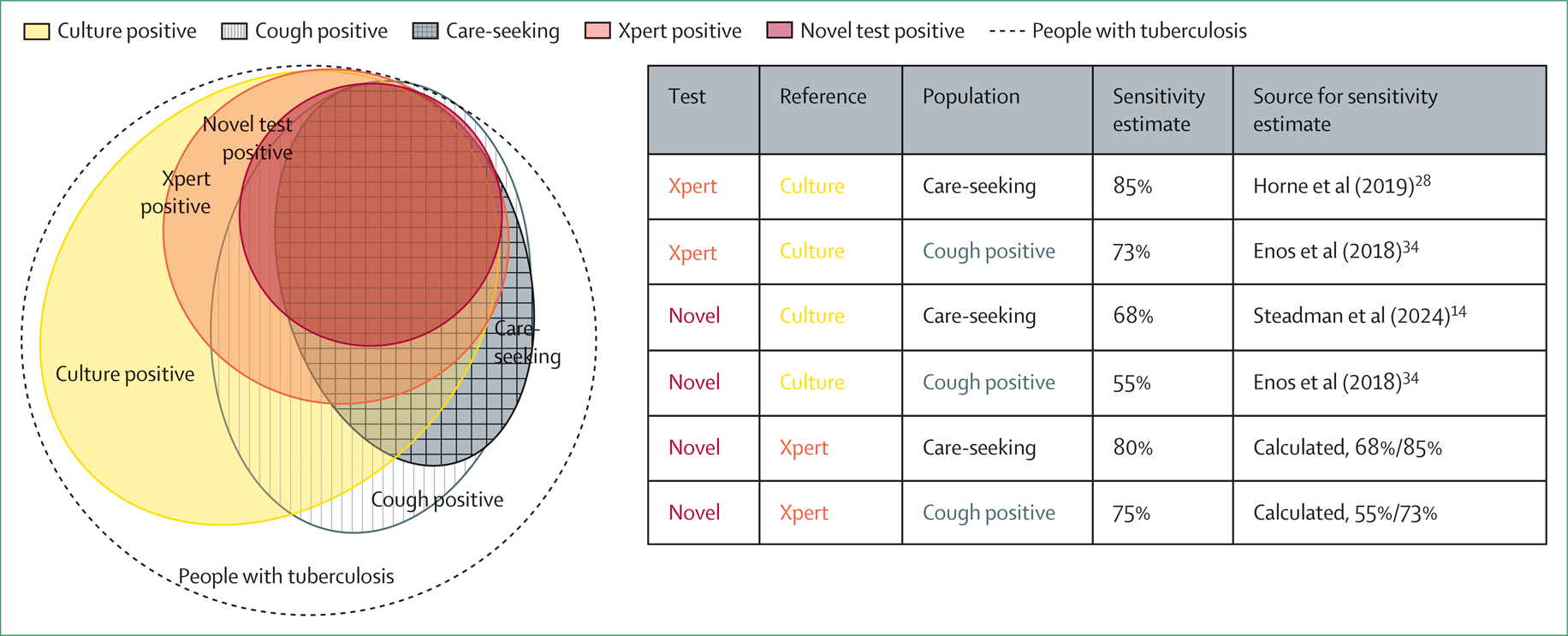
Test sensitivity is a function of population, test, and reference Data from a national tuberculosis prevalence survey in Kenya^34^ were used to estimate the sensitivity of Xpert and a hypothetical novel sputum test with a higher limit of detection than that of sputum culture, among all individuals who screened positive for cough. The novel test is a hypothetical test assumed to have a limit of detection similar to that of an Xpert semiquantitative result classified as medium. Data from diagnostic accuracy studies^14,28^ were used to estimate sensitivities of the same tests in care-seeking patient populations (data from epidemiologically similar settings were preferred where possible). The resulting Venn diagram illustrates that different selection criteria for testing, such as testing only care-seeking patients at a clinic or testing all individuals in the population who screen positive for cough, yield different subsets of individuals with tuberculosis for testing. Additionally, bacteriological reference standards (eg, culture or Xpert) identify only subsets of all tuberculosis cases. Sensitivity estimates using an Xpert reference standard are calculated as a ratio of the sensitivities of the test and the Xpert reference, each relative to culture, in the target population. Due to correlations between bacteriological burden and clinical manifestations of tuberculosis, and between different bacteriological measures, a novel test is estimated to show higher sensitivity when evaluated in a more symptomatic population or against a less sensitive reference standard. Xpert=Xpert MTB/RIF Ultra.

Figure 3 illustrates the dependence of sensitivity estimates on both population and reference standards. We consider a hypothetical novel test (modelled on a low-cost, point-of-care tongue swab) that is optimised for accessibility rather than sensitivity and is assumed to detect *M tuberculosis* in the respiratory tract at levels corresponding to a medium or higher semiquantitative sputum Xpert result. Figure 3 shows, within the overall population with tuberculosis, the subsets detectable by the novel test, sputum Xpert, and sputum culture (represented by larger overlapping circles). These subsets are overlaid onto the overall population with tuberculosis along with the groups that would undergo testing under two possible selection processes: population-wide screening for any cough or testing only those individuals who seek care for (generally severe) symptoms. Based on estimates informed by diagnostic accuracy studies and a prevalence survey,^14,28,34^ the novel test detects only 33% of all culture-positive tuberculosis in the general population. Nonetheless, as respiratory tract bacterial burden (the basis for detection) is correlated with symptoms, the novel test has a substantially higher sensitivity for culture-positive tuberculosis among individuals who screened positive for cough (55%) and even higher sensitivity among those seeking care for symptoms (68%). Figure 3 also illustrates that the sensitivity of the novel test is lower when estimated relative to culture than when estimated relative to Xpert as the reference standard (68% *vs* 80% among individuals seeking care for symptoms) and would be even lower if compared with a more comprehensive reference. Thus, if the test were used for screening people seeking care for symptoms (with positive results confirmed by Xpert), its sensitivity in this context could be 80%, rather than the 33% estimated relative to culture across the entire population.

## Population selection and test correlations affect specificity

Test specificity also depends on the population tested and correlations with other tests. There are several reasons for a tuberculosis screening or diagnostic test to be positive in a person who does not have tuberculosis. Although some reasons for such results (eg, laboratory contamination or error) might not involve the individual being tested, most can be interpreted as appropriate results within a broader spectrum not limited to tuberculosis. For example, when using x-ray, an inflammatory marker, or a sputum molecular test, results classified as falsely positive for tuberculosis (ie, results that misclassify tuberculosis status, even if the assay measures its target accurately) might reflect a high degree of pulmonary pathology, systemic illness unrelated to tuberculosis, or prior tuberculosis disease or exposure.

**Figure 4:**
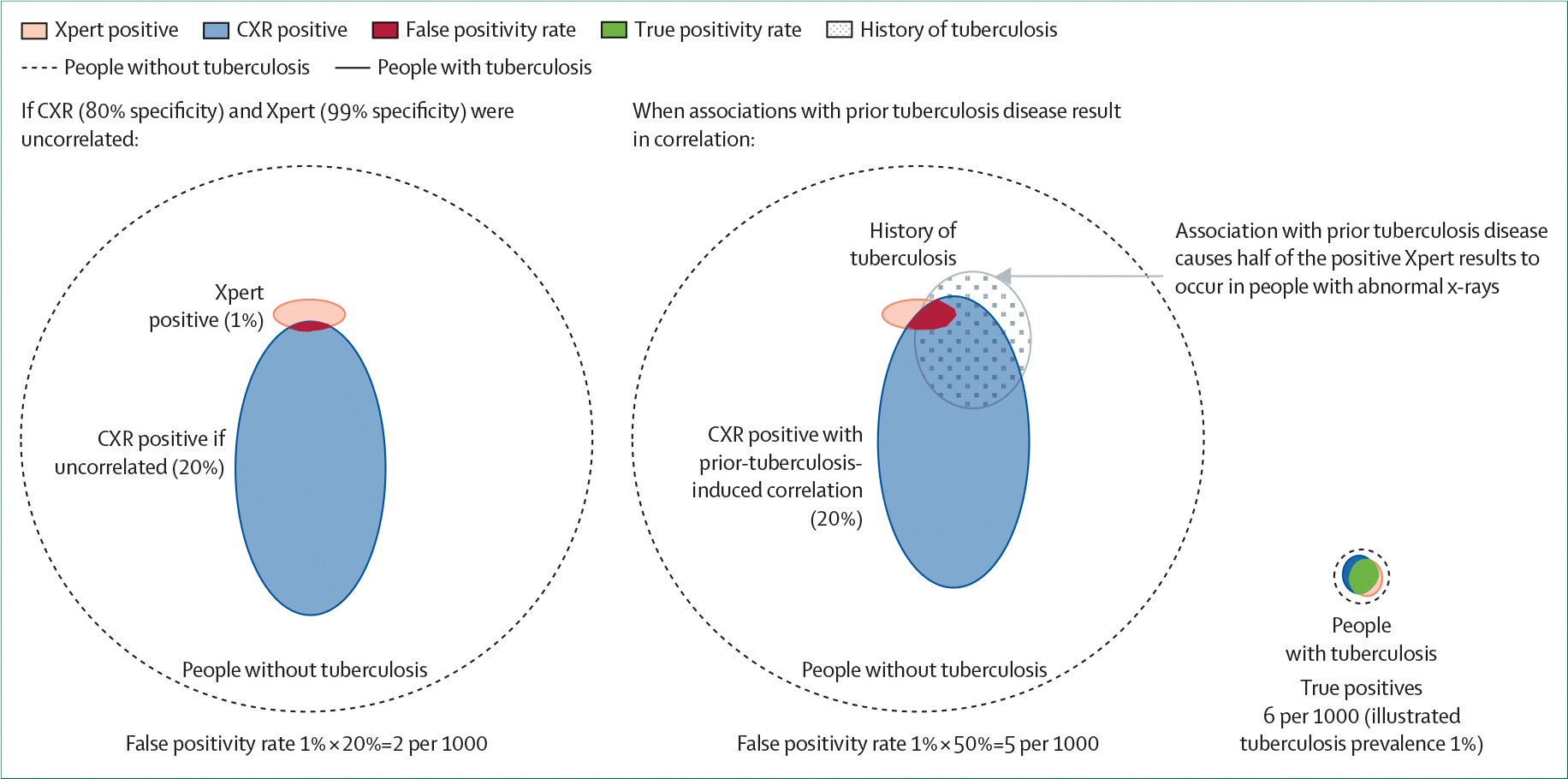
Correlations between tests influence specificity The large circles represent all individuals without tuberculosis who undergo a hypothetical intervention of screening CXR followed by a confirmatory sputum molecular test (Xpert), assuming individual specificity of 80% for CXR and 99% for Xpert in the screened population. Individuals with tuberculosis (at a population prevalence of 1%) are indicated by a small, dotted circle (green) to the right for comparison. If the two tests were independent (leftmost panel), 20% of all positive Xpert results in individuals without tuberculosis would occur among people with abnormal CXR findings, resulting in false-positive tuberculosis diagnoses in (1–80%) × (1–99%)=0·2%, or two per 1000 people evaluated with this algorithm (shown in red in first panel). However, positive results are often correlated across tests owing to underlying individual characteristics. In this example, previously resolved tuberculosis might cause both abnormal CXR findings and positive sputum molecular test results in individuals who do not have active tuberculosis disease. Such a correlation between the two tests leads to 50% (rather than 20%) of positive Xpert results occurring in individuals with abnormal x-ray findings (middle panel) and increases the number of false-positive tuberculosis diagnoses by a factor of 2·5, to five per 1000 individuals evaluated with the algorithm. In a screening context, the absolute number of false-positive tuberculosis results (red) may be large relative to the number of true-positive tuberculosis diagnoses (green). CXR=chest x-ray. Xpert=Xpert MTB/RIF Ultra.

The mechanisms underlying so-called false positivity are often correlated across different tests. For example, non-tuberculosis lung infections can simultaneously cause respiratory symptoms, abnormal chest x-ray findings, and elevated C-reactive protein levels. The effects of correlation on the specificity of multitest algorithms are analogous to its influence on sensitivity within the tuberculosis disease spectrum: correlation improves the combined specificity of concurrent testing (because fewer people without tuberculosis have any positive result) but is detrimental to the specificity of sequential screening algorithms (because of increased overlap of positive results on screening and confirmatory testing steps among individuals without tuberculosis). These findings are illustrated in figure 4 for a typical two-step screening algorithm of chest x-ray followed by molecular testing. A history of treated or resolved tuberculosis might increase the probability of a positive result on the screening chest x-ray (due to post-tuberculosis lung disease)^35^ and on the confirmatory tuberculosis molecular test (owing to residual DNA in sputum specimens).^36^ Thus, previous tuberculosis disease induces a correlation between the specificities of the screening and confirmatory steps, resulting in lower combined specificity than would be expected if the two tests were independent.

The mechanisms underlying false positivity for tuberculosis vary across populations, resulting in corresponding variations in test specificity. For example, among culture-negative individuals, Xpert Ultra yields more positive results in care-seeking patients than in the general population.^37,38^ A possible explanation is that sources of Xpert positivity (eg, previously resolved tuberculosis) are associated with respiratory symptoms. Thus, applying clinic-based specificity estimates to lower-prevalence settings might lead to a smaller reduction in negative predictive value than would be expected based on prevalence alone.^38,39^ Therefore, accurate estimation of the specificity of algorithms requires either directly measuring correlations between the specificities of individual tests in the relevant testing population or characterising the sources of positive results within the population of interest and estimating the joint specificity accordingly.

## Implications and recommendations

Estimates of the sensitivity and specificity of tuberculosis screening and diagnostic tests depend on the extent to which multiple testing steps and patient-selection processes align along one or more dimensions of an underlying disease spectrum. Even for tests used in isolation, sensitivity depends on the ability of the tests to detect the segments of the tuberculosis disease spectrum that are included in both the testing and reference populations. For algorithms involving two or more tests, sensitivity additionally depends on the degree to which the tests detect overlapping segments of the disease spectrum. Sensitivities might differ for high-priority subgroups compared with the overall population with tuberculosis. Similarly, the specificity of multitest algorithms depends on the mechanisms underlying positivity, their prevalence in the tested population, and the extent of overlap in these mechanisms across tests. These spectrum and context dependencies have important implications for the evaluation, reporting, and modelling of tuberculosis diagnostic test accuracy, as summarised in the panel.

First, the patients included in diagnostic accuracy studies tend not to be representative of general populations. Therefore, study designs should facilitate translation of findings to other populations that might, for example, have fewer symptoms, reduced access to health care, or face difficulty in producing sputum. This translation can be aided by characterising how the performance of a test relates to individual characteristics and results of other tests. For tests that can be used together in practice, concurrent evaluation in the same individuals provides the clearest insights into test interactions and can inform the design of testing algorithms whose test combinations and selected cutoff values optimise accuracy and resource efficiency.^22^ Importantly, characterising the disease in the study population along multiple dimensions can aid in translating results across potential populations and testing algorithms. The translation of results can be achieved by evaluating novel tests alongside a small number of established, well-characterised, low-cost tests (ie, comparator tests) that reflect disease dimensions such as bacterial load and clinical severity. Results should be recorded on quantitative scales where possible (eg, Xpert cycle threshold or liquid culture time to positivity for bacterial load; quantitative C-reactive protein for host inflammatory response; and computer-aided detection scores for radiographic extent), to allow exploration of different diagnostic cutoff values and facilitate more granular mapping across patient populations. Tests should be evaluated head-to-head in the same individuals, at the same time, and ideally on the same sample.

Second, to maximise the value of diagnostic studies, researchers should report results with greater granularity than is typically provided. For example, accuracy estimates could be stratified by indicators such as disease severity and *M tuberculosis* bacterial load and presented using multiway tables or subgroup analyses that reflect likely test combinations and use cases. This enhanced level of detail would facilitate comparisons across studies, support the translation of findings to new populations and testing algorithms, and enable evaluation of tests against reference standards, such as sputum molecular testing, that are less sensitive than culture-based and composite reference standards but might better reflect pragmatic use. Additionally, providing individual-level data at the time of publication would allow for reweighting based on multiple disease indicators when extrapolating results, thereby offering greater flexibility and value for subsequent research and applications.

Third, when establishing and communicating accuracy targets (for example, in TPPs), experts and policy makers should explicitly consider the disease spectrum by clearly defining the intended testing scenarios and target populations. For instance, a screening test reported as 90% sensitive can have different interpretations: it could detect 90% of all prevalent tuberculosis cases, 90% of tuberculosis cases detectable by existing screening tests (eg, individuals who are symptomatic or x-ray positive), or 90% of tuberculosis cases that would test positive in a confirmatory step—the metric most relevant to combined sensitivity when the test is part of a stepwise algorithm. In addition, TPPs might specify separate accuracy targets for high-priority subgroups to guide the development of novel tests that add incremental value. These high-priority subgroups could include individuals with high bacillary burdens, groups with high-risk comorbidities, or populations in whom tuberculosis is difficult to diagnose definitively, such as children. For tests intended for use in multitest algorithms, high-priority subgroups could also be defined in relation to other tests—for instance, by prioritising true-positive results that would be confirmed in sequential algorithms or add incremental positivity in concurrent algorithms. For a screening test that will be followed in practice by a molecular test less sensitive than culture, TPPs should consider setting sensitivity targets relative to the same less sensitive test, indicating not only that the screening test should achieve least 70% sensitivity for all tuberculosis cases but also that it should detect at least 90% of sputum molecular test-positive cases. The most relevant reference standard is not necessarily the broadest, but rather the one that aligns with expected use or identifies target populations of high clinical or public health relevance.

Finally, people interpreting diagnostic accuracy data, including modellers of diagnostic interventions, should exercise caution when directly transferring accuracy estimates across populations or assuming that different tests operate independently. When empirical data are unavailable for a population or testing context of interest, accuracy estimates from other settings can be adapted by accounting for differences in key covariates (eg, prevalence of symptoms, presence of comorbidities, or level of bacterial burden, depending on the mechanism of the test) that are likely to affect performance. Furthermore, when modelling diagnostic algorithms, it is important to account for test correlations. One approach is to represent tests stepwise and use empirical data to estimate sensitivity and specificity within subpopulations defined by the results of earlier testing steps.

No approach is without limitations, and explicitly addressing the complexities of tuberculosis diagnostics can lead to algorithms with improved performance. As a final illustration, tongue swab-based molecular tests represent a new point-of-care tool that could be incorporated into active case-finding efforts, as reported in a preprint.^18^ When evaluated against a culture-based reference in a general population, the sensitivity of tongue swab assays is likely to be suboptimal—although greater than that of sputum smear microscopy—for the conventional screening role of confidently ruling out disease. Furthermore, specificity might appear diminished when evaluated in symptomatic care-seeking populations.^40^ Nevertheless, these tests could offer high value in several possible screening algorithms. As the first step in a two-step algorithm in which positive tongue swab results are confirmed by sputum Xpert or another tongue swab, tongue swab screening would offer good correlation with the confirmatory test, thereby maximising the sensitivity of the algorithm despite suboptimal sensitivity of each component test. Additionally, tongue swab screening would also offer high sensitivity for highly infectious forms of tuberculosis,^41^ good affordability, and increased accessibility. These advantages might be prioritised over sensitivity relative to culture, and evaluations of tongue swab-based testing should account for this prioritisation. Alternatively, tongue swabs could provide value as a confirmatory test in algorithms with chest x-ray and a quantitative computer-aided detection score readout as the screening step. In this role, tongue swabs would confirm most tuberculosis cases detectable by sputum Xpert, eliminate the need for sputum production (potentially increasing the overall diagnostic yield), and among individuals with low-positive computer-aided detection scores, provide adequate negative predictive value despite their modest sensitivity. Meanwhile, for enhancing algorithm sensitivity, individuals with the highest quantitative computer-aided detection scores could be prioritised for sputum Xpert testing—either immediately or after a negative tongue swab result. A third option for tongue swabs would be to use them as standalone tests for active case finding. Screening algorithms usually require at least two steps to ensure adequate combined specificity. However, given the expected high specificity in the screening context,^39^ a molecular test that is affordable for universal use might also provide adequate positive predictive value for standalone screening in high-burden populations.

## Conclusion: putting tests in context to maximise their impact

The goal of developing new diagnostic assays is to add incremental clinical and public health value. To achieve this goal, tests need to be accurate, and their accuracy should be evaluated and interpreted within the context in which they will be used, including the epidemiological setting and diagnostic algorithms. Particularly for tuberculosis screening, in which tests are applied across a broad disease spectrum and within multistep algorithms, context-sensitive consideration of accuracy requires understanding how tests interact with multiple dimensions of the disease spectrum. Key aspects include clearly specifying the population and reference standard in accuracy estimates, considering and modelling how correlations affect algorithm accuracy, defining accuracy targets in ways that align with the test’s expected use, and designing studies to generate and report high-quality data on test correlations and interactions to support population-specific and algorithm-specific estimation of accuracy. These actions can facilitate the development of more efficient and impactful tuberculosis diagnostic algorithms, helping to close diagnostic gaps and reduce the global burden of tuberculosis.

## *Panel:* Implications of diagnostic test correlations for the measurement and evaluation of test accuracy in tuberculosis

### Recommendations for performing diagnostic accuracy studies

#### Challenge: Test performance might reflect the population tested

- Pair novel assays with well established assays (eg, Xpert cycle threshold or CRP) that can characterise disease severity and delineate detection thresholds along relevant dimensions
- Characterise sources and correlates of positivity (eg, other inflammatory or respiratory conditions or prior tuberculosis disease) among individuals without tuberculosis in the study population

#### Challenge: Algorithm performance depends on interactions among tests

- For tests likely to be combined (in concurrent or stepwise algorithms), evaluate concurrently in the same individuals
- Use continuous scales (eg, molecular test cycle threshold, chest x-ray abnormality score) where possible to allow exploration of different cutoffs

### Recommendations for reporting diagnostic accuracy studies

#### Challenge: Accuracy estimates require context-specific interpretation

- Detail the clinical setting and the presence or severity of symptoms
- Specify eligibility or screening criteria

#### Challenge: Data can help to translate accuracy estimates to new populations

- Stratify results by comparator tests (eg, Xpert or CRP)
- Make individual-level data available

#### Challenge: Test correlations affect algorithm accuracy

- Report the sensitivity of screening tests relative to likely confirmatory tests
- Report the incremental sensitivity of diagnostic tests relative to other tests likely to be used concurrently
- Report correlations in specificity between tests

### Recommendations for setting accuracy targets (eg, in target product profiles)

#### Challenge: Numerical accuracy can vary by context

- Specify the intended testing setting and target population for numerical accuracy targets
- Specify the intended reference standard, particularly for tests targeting culture-negative tuberculosis or those confirmed in practice by relatively low-sensitivity tests

#### Challenge: Diagnostic objectives extend beyond simple accuracy

- Consider setting dedicated sensitivity targets for forms of tuberculosis that pose the highest clinical or transmission risk; will also be positive on other tests used sequentially; and would be missed by other tests used concurrently

### Recommendations for choosing tests and modelling performance

#### Challenge: Sensitivity and specificity vary across populations

- Prioritise data from contexts similar to the context of interest
- Reweigh based on other correlated tests or characteristics when extrapolating results from other contexts

#### Challenge: Tests that are combined into algorithms often have correlated sensitivity and specificity

- Use empirical data from simultaneous testing when available
- When appropriate, adjust for accuracy relative to spectrum benchmarks such as markers of bacterial load or clinical severity, depending on the test mechanism

#### Challenge: Diagnostics should add clinical and public health value

- Estimate health-relevant outcomes beyond the proportion of cases detected (eg, incremental detection and the morbidity, mortality, or transmission averted)

Xpert=Xpert MTB/RIF Ultra. CRP=C-reactive protein.
